# Transplantation of PSC-derived myogenic progenitors counteracts disease phenotypes in FSHD mice

**DOI:** 10.1038/s41536-022-00249-0

**Published:** 2022-09-02

**Authors:** Karim Azzag, Darko Bosnakovski, Sudheer Tungtur, Peter Salama, Michael Kyba, Rita C. R. Perlingeiro

**Affiliations:** 1 Lillehei Heart Institute, Department of Medicine, University of Minnesota, Minneapolis, MN USA; 2 Lillehei Heart Institute, Department of Pediatrics, University of Minnesota, Minneapolis, MN USA; 3 Stem Cell Institute, University of Minnesota, Minneapolis, MN USA

**Keywords:** Pluripotent stem cells, Diseases

## Abstract

Facioscapulohumeral muscular dystrophy (FSHD) is a genetically dominant progressive myopathy caused by improper silencing of the DUX4 gene, leading to fibrosis, muscle atrophy, and fatty replacement. Approaches focused on muscle regeneration through the delivery of stem cells represent an attractive therapeutic option for muscular dystrophies. To investigate the potential for cell transplantation in FSHD, we have used the doxycycline-regulated iDUX4pA-HSA mouse model in which low-level DUX4 can be induced in skeletal muscle. We find that mouse pluripotent stem cell (PSC)-derived myogenic progenitors engraft in muscle actively undergoing DUX4-mediated degeneration. Donor-derived muscle tissue displayed reduced fibrosis and importantly, engrafted muscles showed improved contractile specific force compared to non-transplanted controls. These data demonstrate the feasibility of replacement of diseased muscle with PSC-derived myogenic progenitors in a mouse model for FSHD, and highlight the potential for the clinical benefit of such a cell therapy approach.

## Introduction

Facioscapulohumeral muscular dystrophy (FSHD) is an autosomal dominant muscle disorder with a conservative incidence of 1 in 15,000 births worldwide^1,2^, representing the third most common inherited muscular dystrophy, following Duchenne muscular dystrophy (DMD) and myotonic dystrophy (DM). The most affected muscles are typically the facial, shoulder, and forearm muscles; however, the disease shows high variability between patients, as well as asymmetric presentation within individual patients, with most skeletal muscles being affected to some degree. The diaphragm is usually largely spared and cardiac muscle does not show evidence of degeneration, thus although FSHD can be tremendously debilitating, patients experience normal lifespans and the medical burden of FSHD is large.

FSHD is caused by the defective epigenetic repression of the D4Z4 macrosatellite repeat array at chr4qter^3–6^. Each unit of this array contains an ORF encoding DUX4^7^, a transcription factor with two homeodomains that bind DNA in a head-to-head fashion^8^. Incomplete repression, commonly observed as decreased DNA methylation^9–12^ leads to transcriptional leakage in muscles of patients^9^. The levels of leakage are very low, and direct immunodetection of DUX4 in muscles of patients is lacking, although weakly detectable increased expression of known DUX4 target genes in FSHD muscle biopsies^10^ implies that DUX4 is or was expressed there.

DUX4 may normally play some role in embryonic development^11–16^, but its expression at later times is thought to be deleterious. In vitro, DUX4 expression leads to cell death^17,18^. DUX4-expressing mouse models have been generated to study FSHD pathology and for testing therapeutics^19–24^. Two inducible strategies have been reported to control DUX4 toxicity in somatic tissues: cre-^23,24^ and doxycycline (dox)-inducible models^21,22^. One useful feature of the later approach is the dual ability to turn DUX4 expression both on and off. Successful modulation of DUX4 expression has been reported in the iDUX4pA-HSA mouse, the first published model to show loss of muscle due to DUX4 expression, in which the human skeletal actin (HSA) promoter drives expression of rtTA, thus enabling dox-regulated DUX4 expression in skeletal muscle fibers^22^. This iDUX4pA-HSA mouse model recapitulates several disease phenotypes observed in FSHD patients, including inflammation, fibrosis, and muscle atrophy, among others, and therefore, it has been used with success to test therapeutics^22,25^.

Although at present, only palliative treatments, aiming to slow down disease progression and improve quality of life, are approved for FSHD^26^, there have been several recent clinical trials targeting disease processes. The p38 inhibitor Losmapimod, recently tested in a Phase 2 clinical trial for FSHD patients (ClinicalTrials.gov Identifier: NCT04003974) has been shown to decrease the expression of DUX4 and its target genes in a mouse xenograft model^27^. Other clinical trials are currently evaluating the benefits of drugs approved for other diseases in the context of FSHD, such as the beta-2 receptor agonist albuterol (ClinicalTrials.gov Identifier: NCT00027391), the myostatin inhibitor ACE-083 (ClinicalTrials.gov Identifier: NCT02927080), and the creatine monohydrate ATYR1940 (ClinicalTrials.gov Identifier: NCT02603562). To date, none of these have met their primary endpoints. Other approaches targeting DUX4 have shown some promise in preclinical studies. For instance, successful reduction of DUX4 mRNA levels has been reported using siRNA, miRNA, and antisense oligonucleotide direct against DUX4^28–35^. Another potential target is a G-quadruplex, present within D4Z4^36^. Berberine, a natural G-quadruplex ligand, has the ability to reduce DUX4 mRNA levels^37^. DUX4 cofactor inhibitors have also been considered targets for FSHD. DUX4 interacts with the histone acetyltransferases p300 and CBP^38^ and a p300 inhibitor has been tested in iDUX4pA-HSA mice, which resulted in a reduction of DUX4 target gene expression^22,25^.

Another potential therapeutic approach is cell replacement, in which the diseased muscle is replaced with healthy myofibers, and ideally, new healthy muscle stem cells. This strategy has been extensively investigated in the context of other types of muscular dystrophy^39,40^. Among the different cell types under investigation, pluripotent stem cells (PSCs) are an attractive source since they can continually produce large amounts of differentiated tissue. Transplantation of PSC-derived myogenic progenitors has proven effective in several models of recessive muscular dystrophy, including DMD, for which mdx and *mdx/Utrn*^−/−^ double knockout mice have been tested^41–45^, LGMD2D^46^, LGMD2A^47^, and LGMD2I^48,49^.

To determine the therapeutic potential of PSC-derived myogenic progenitors in FSHD, we took advantage of the iDUX4pA-HSA mouse model referred to above, in which dox treatment allows for the conditional expression of human DUX4 specifically in skeletal muscle^22^.

Here we show that mouse PSC-derived myogenic progenitors have the ability to promote muscle regeneration in the presence of an FSHD-like environment, namely muscle undergoing DUX4-mediated degeneration, as shown by the presence of donor-derived myofibers and satellite cells. Importantly, engrafted muscles showed improved muscle strength and reduced DUX4-induced fibrosis. These observations establish the proof of concept for the use of cell transplantation as a suitable therapeutic intervention for FSHD.

## Results

### Establishing cell transplantation in the iDUX4pA-HSA mouse model

**Fig. 1:**
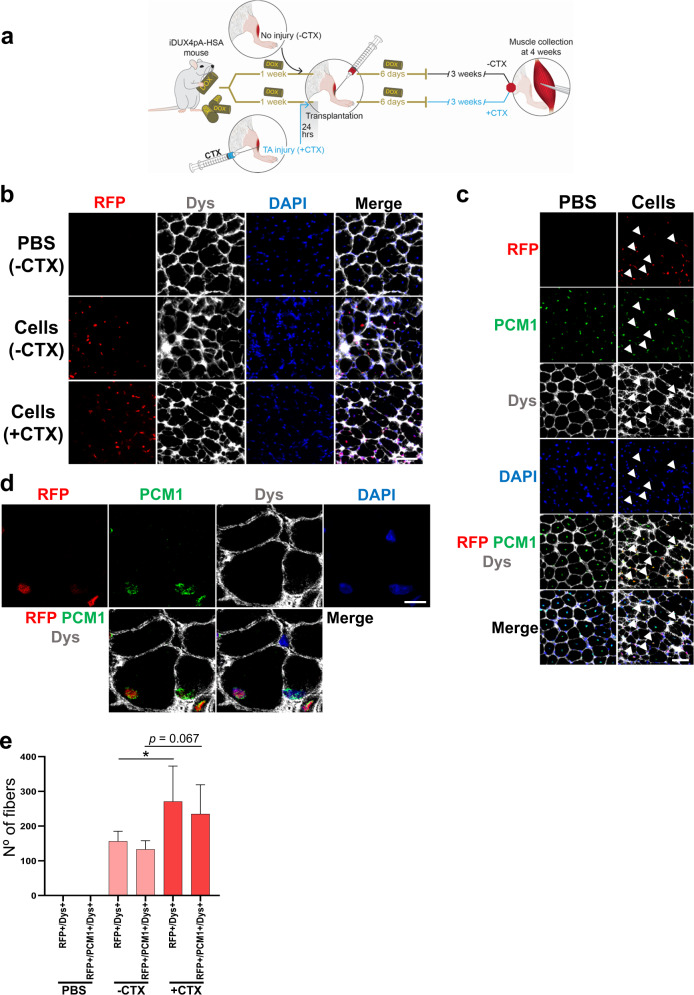
In vivo regenerative potential of Pax3-induced myogenic progenitors in iDUX4pA-HSA mice. **a** Outline of experimental design. **b** Representative images show immunostaining for RFP (in red) and Dys (in gray) in non-injured (−CTX) and CTX-injured (+CTX) TA muscles from iDUX4pA-HSA mice that had been transplanted with Pax3-induced mouse ESC-derived myogenic progenitors. Images from PBS-injected TA muscle are shown in the upper panel (-CTX). DAPI stains nuclei (in blue). Scale bar is 50 µm. **c** Representative images showing immunostaining for RFP (in red), PCM1 (in green) and Dys (in gray) in TA muscles of iDUX4pA-HSA mice that had been injected with PBS (left panel) or cells (right panel) from the +CTX group. DAPI stained nuclei in blue. Arrowheads show RFP +/PCM1 + nuclei. Scale bar is 50 µm. **d** Representative confocal images from +CTX group show a nucleus positive for RFP and PCM1. Scale bar is 10 µm. **e** Engraftment quantification based on the number of RFP + /Dys+ and RFP + /Dys + /PCM1 + myofibers. Data are shown as mean ± SEM (*n* = 6 for PBS, *n* = 6 for -CTX and *n* = 4 for +CTX). *p < 0.05 by the Student’s *t* test.

To determine the muscle regenerative potential of PSC-derived myogenic progenitors in the context of DUX4-mediated muscle degeneration, as occurs in FSHD, we transplanted Pax3-induced murine embryonic stem cell (ESC)-derived myogenic progenitors into iDUX4pA-HSA mice. DUX4 expression was induced by feeding mice dox chow^50^, which began 7 days prior to transplantation, and was maintained for 6 days thereafter (Fig. 1a). Dox-induced iDUX4pA-HSA mice were subdivided into 2 groups, one cohort in which *tibialis anterior* (TA) muscles were pre-injured with cardiotoxin (CTX) and another cohort that remained uninjured (Fig. 1a). CTX injury is widely used for muscle engraftment assessment since it destroys existing muscle fibers, initiating a generalized regenerative program, allowing the efficient contribution of transplanted cells to newly regenerated muscle^41,51–53^. Of note, it has been reported previously that new fiber formation after CTX injury is negligible in the presence of maintained DUX4 induction in the iDUX4pA-HSA model^22^, suggesting that this experimental setting may provide an environment that is free of host competition after induction of injury. To detect the presence of donor-derived myofibers, transplanted cells carried an integrated H2B-RFP fusion protein driven by the PGK promoter, which we have previously demonstrated to give robust expression in myofibers^48,54^, while avoiding the dilution observed with cytoplasmic GFP, which can create difficulties given muscle autofluorescence^55^. One month post-transplantation, we observed donor-derived myofibers in both cohorts of iDUX4pA-HSA mice. We confirmed donor identity by the presence of RFP positive (RFP+) nuclei and employed dystrophin staining to ensure that RFP + nuclei were in fact within a muscle fiber (Fig. 1b). We validated the position of the RFP + nuclei inside the myofiber and the subsequent quantifications using a specific marker for myonuclei, PCM1^56,57^ (Fig. 1c, d). Both readouts revealed higher levels of donor engraftment in iDUX4pA-HSA mice that had been pre-injured with CTX (Fig. 1e). Therefore, all subsequent studies employed CTX pre-injury.

### Optimizing DUX4-mediated injury for assessing regenerative potential

**Fig. 2:**
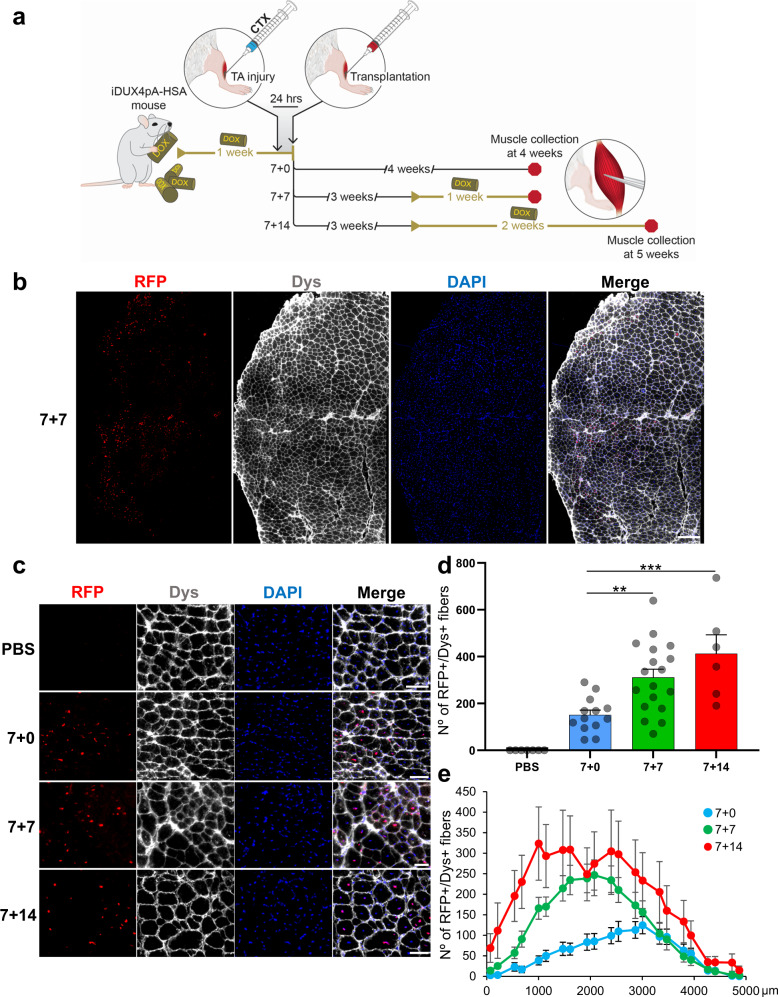
Engraftment is persistent even in the context of bursts of DUX4 expression. **a** Outline of experimental design. **b** Representative images, capturing the complete engrafted area, show immunostaining for RFP (in red), Dys (in gray), and DAPI (in blue) of the 7 + 7 cohort. Scale bar is 200 µm. **c** Representative images show the same staining as **b** for the 3 experimental groups depicted in **a**: 7 + 0, 7 + 7, and 7 + 14. Upper panel shows PBS-injected control. Scale bar is 50 µm. **d** Graph shows quantification of engraftment (from **c**) as shown by the number of donor-derived RFP + /Dys+ myofibers. Data are shown as mean ± SEM (*n* = 7 for PBS, *n* = 13 for 7 + 0, *n* = 18 for 7 + 7 and *n* = 6 for 7 + 14). **p < 0.01, ***p < 0.001 by the Student’s *t* test. **e** Distribution of the number of RFP + /Dys+ myofibers along the TA muscle. Data are shown as mean ± SEM (*n* = 13 for 7 + 0, *n* = 18 for 7 + 7, and *n* = 6 for 7 + 14).

The distinguishing feature of the iDUX4pA-HSA mouse model is the ability to turn off DUX4 expression with dox withdrawal, allowing pulses or bursts of DUX4 expression^22^. To better understand the behavior of engrafted donor-derived myofibers in the presence of DUX4, we designed a series of transplantations in which iDUX4pA-HSA mice were exposed to dox continually (Supplementary Fig. 1a) or over different time windows (Fig. 2a). For continual DUX4 induction, recipient mice were fed with dox chow during the whole course of the experiment (Supplementary Fig. 1a). Our data showed that continual dox induction was not compatible with the in vivo differentiation of transplanted Pax3-induced myogenic progenitors into myofibers. Continued dox leads to constant Pax3 expression, which prevents the differentiation of injected myogenic progenitors into donor-derived myofibers, as cells in this arm remained Pax3+ throughout the study (Supplementary Fig. 1b, c). For the time window studies, iDUX4pA-HSA mice were exposed to dox for 7 days prior to transplantation, which is sufficient to induce the DUX4-dystrophic phenotype (Supplementary Fig. 2). They were then allowed to regenerate for 3 weeks in the absence of DUX4 induction, and then given a second pulse of DUX4 for different lengths of time, as outlined in Fig. 2a (7 + 0, 7 + 7, and 7 + 14). As above, donor-derived myofiber engraftment was identified by immunofluorescence staining at the point of muscle collection (Fig. 2b, c). Both fiber quantifications, RFP + /Dys + (Fig. 2d) and RFP + /Dys + /PCM1 + (Supplementary Fig. 3a–c), showed the highest levels of engraftment in iDUX4pA-HSA mice that were re-exposed to DUX4, with an average of 400 donor-derived myofibers per TA muscle in the 7 + 14 cohort, which represents approximately 15% of the total muscle cryosectional area (Supplementary Fig. 4a, b). Assessment of RFP + donor-derived myofiber distribution along engrafted TA muscles showed that transplanted cells were robustly distributed along the TA muscle (Fig. 2b, e). We also detected the presence of donor-derived satellite cells, as indicated by the identification of Pax7 + /RFP + cells under the basal lamina (Supplementary Fig. 5a). Quantification showed that the frequency of Pax7 + /RFP + cells was proportional to the degree of myofiber engraftment, with up to 9% of donor-derived satellite cell engraftment detected in the groups 7 + 7 and 7 + 14 (Supplementary Fig. 5b).

**Fig. 3:**
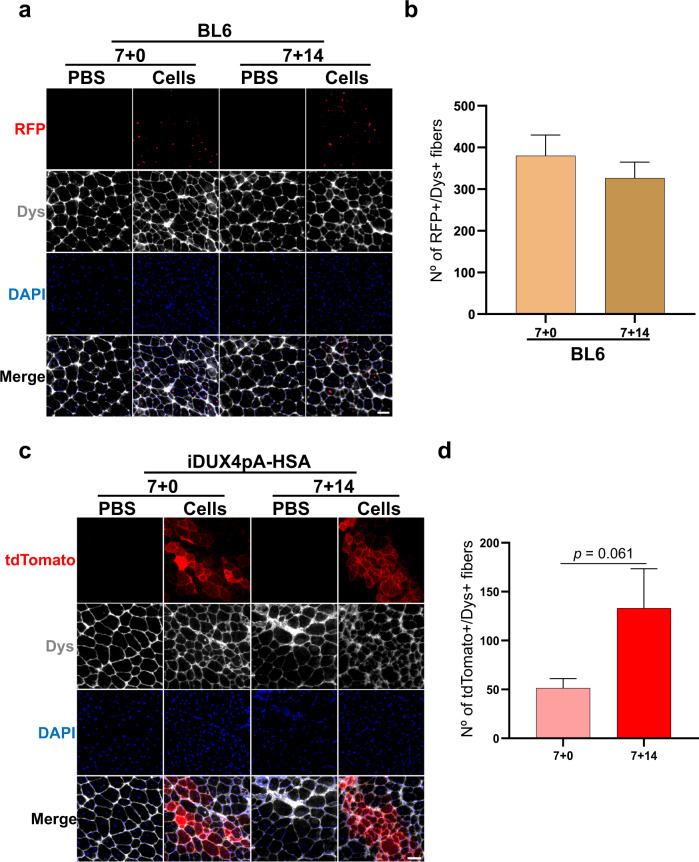
Testing environment vs. cell autonomous effect of dox post-transplant. **a** Representative images show immunostaining for RFP (in red) and Dys (in gray) in TA muscles of BL6 mice that had been injected with PBS or Pax3-induced myogenic progenitors, and later fed with dox in a similar fashion as iDUX4pA-HSA 7 + 0 and 7 + 14 cohorts (Fig. 2a). DAPI stained nuclei in blue. Scale bar is 50 µm. **b** Graph shows engraftment quantification from **a**. Data are shown as mean ± SEM (*n* = 7 for 7 + 0 and *n* = 7 for 7 + 14). **c** Representative images show immunostaining for tdTomato (in red), and Dys (in gray) in TA muscles of iDUX4pA-HSA mice that had been injected with PBS or 5000 satellite cells isolated from a mTmG mouse and fed with dox following the 7 + 0 and 7 + 14 dox regimen. DAPI stained nuclei in blue. Scale bar is 50 µm. **d** Graph shows engraftment quantification from **c** based on the number tdTomato + /Dys+ myofibers. Data are shown as mean ± SEM (*n* = 6 for 7 + 0 and *n* = 5 for 7 + 14). *p* = 0.061 by the Student’s *t* test.

To determine whether Pax3 re-expression due to the second pulse of dox treatment may have contributed to improved engraftment, we transplanted Pax3-induced myogenic progenitors into non-DUX4 mice, both immunosuppressed wild-type (WT) C57BL/6 (BL6) and NSG mice, following the same dox treatment shown in Fig. 2. Donor-derived myofibers were detected in all groups, but there was no increase in engraftment in mice that were treated with dox after transplantation (7 + 14) compared to control (7 + 0) recipients (BL6 shown in Fig. 3a, b; NSG shown in Supplementary Fig. 6a, b). This suggests that enhanced engraftment in the iDUX4pA-HSA mouse model was entirely due to the additional DUX4-mediated muscle degeneration in the host and not to the re-expression of Pax3 in the donor cells. When we performed the converse experiment, by transplanting a non-Pax3 myogenic cell population, freshly isolated WT satellite cells, into iDUX4pA-HSA mice, we observed donor-derived contribution to muscle regeneration trended higher (*p* = 0.061 by the Student’s *t* test) in the mice that received the second round of DUX4-mediated degeneration (Fig. 3c, d). Taken together, these data suggest that the increased engraftment observed when iDUX4pA-HSA mice receive a second dose of dox is due to DUX4-mediated injury.

### Cell transplantation ameliorates DUX4-mediated fibrosis

**Fig. 4:**
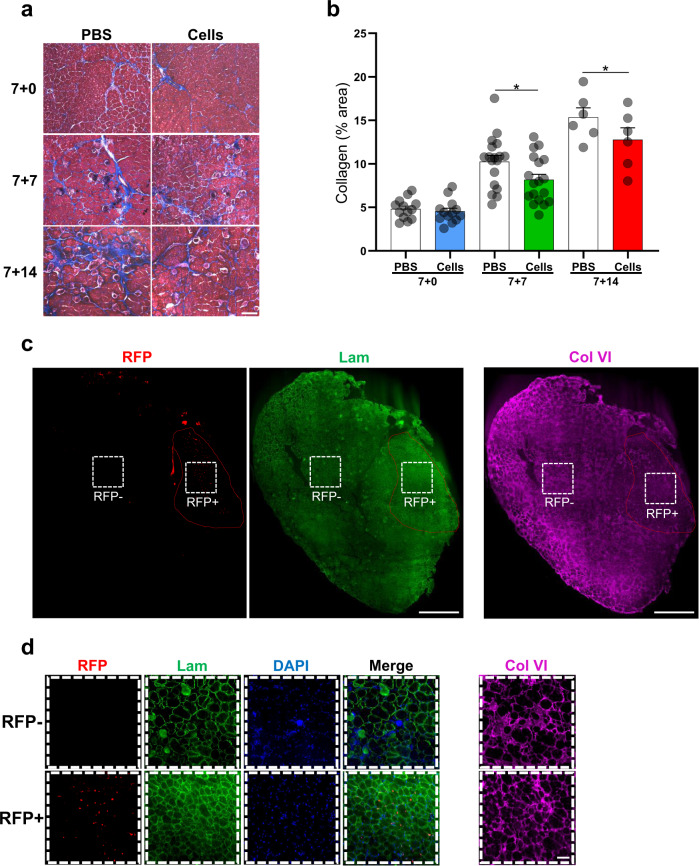
Transplantation of myogenic progenitors counteracts the fibrosis induced by DUX4 expression in iDUX4pA-HSA mice. **a** Representative images show Masson’s trichrome staining for the 3 experimental cohorts of iDUX4pA-HSA mice: 7 + 0, 7 + 7, and 7 + 14. Right panels show transplanted TA muscles, while left panels display respective contralateral PBS controls. Scale bar is 100 µm. **b** Quantification of the percentage of collagen staining in the total muscle section. Data are shown as mean ± SEM (*n* = 13 for 7 + 0, *n* = 18 for 7 + 7 and *n* = 6 for 7 + 14). *p < 0.05 by the Student’s *t* test. **c** Representative images of a whole muscle section show immunostaining for RFP (in red), Lam (in green), and Col VI (in purple; staining performed on the consecutive slide) in transplanted TA muscle from iDUX4pA-HSA mice (7 + 14). Engrafted area has been highlighted in red to denote the engraftment location on the Col VI staining. DAPI in blue. Scale bar is 500 µm. **d** Images show higher magnification of negative and positive RFP areas from **c** (from white dashed lines used to depict RFP- and RFP + areas). Scale bar is 50 µm.

To determine the therapeutic benefit of cell transplantation in iDUX4pA-HSA mice, we investigated the levels of fibrosis, which is a hallmark of this mouse model^22^. The greatest fibrosis was seen in the 7 + 7 and 7 + 14 groups, in accordance with the original publication, which reported about 15% of fibrotic tissue in iDUX4pA-HSA mice following two weeks of dox treatment^22^. Cell transplantation reduced the extent of fibrosis to a statistically significant degree in both the 7 + 7 and 7 + 14 groups, as shown by collagen deposition using Masson’s trichrome staining (Fig. 4a, b). We also evaluated fibrosis within engrafted domains vis-à-vis non-engrafted domains within the same muscle. Immunofluorescence staining for collagen VI (Col VI) revealed reduced fibrosis in the engrafted area compared to the surrounding non-engrafted tissue (Fig. 4c, d and Supplementary Fig. 7). In addition, laminin staining showed that newly engrafted muscle is composed of densely packed fibers, while non-engrafted muscle fibers are more rounded and pushed apart, a consequence of endomysial expansion due to matrix deposition. Furthermore, engrafted domains showed much lower levels of infiltrating cells (observable by 4,6-diamidino-2-phenylindole (DAPI) staining in Fig. 4d). Together, these results show that newly engrafted muscle is less fibrotic and histologically healthier than retained host muscle.

### Functional improvement upon transplantation of myogenic progenitors in iDUX4pA-HSA mice

**Fig. 5:**
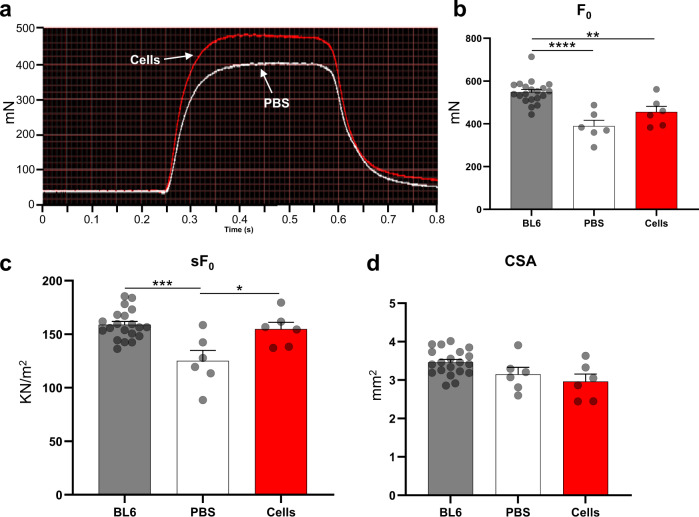
Effect of cell transplantation on the muscle contractile properties of iDUX4pA-HSA mice. **a** Representative example of force tracing upon tetanic stimulation in TA muscles from iDUX4pA-HSA mice. Red and white lines show force tracing from muscles that had received cell transplantation or PBS, respectively. **b**, **c** Effect of cell transplantation on **b** absolute (*F*_0_) and **c** specific (s*F*_0_: *F*_0_ normalized to CSA) force. Control BL6 mice were used as reference. Data are shown as mean ± SEM (*n* = 20 for BL6, *n* = 6 for PBS and cells). *p < 0.05, **p < 0.01, ***p < 0.001, ****p < 0.0001 by the Student’s *t* test. **d** Average CSA of analyzed muscles (from **b**, **c**). Data are shown as mean ± SEM. **p < 0.01 by the Student’s *t* test.

To determine whether engraftment of myogenic progenitors is accompanied by functional improvement of physiological muscle weakness, we performed in situ force measurements. Based on the more severe phenotype, we utilized the 7 + 14 experimental protocol for these studies. When transplanted and control TA muscles were dissected and subjected to electrical stimulation, we found that transplanted muscles displayed significant functional improvements, as demonstrated by both superior isometric tetanic force and specific force when compared to their respective contralateral PBS-injected TA muscles (Fig. 5).

## Discussion

This study investigates the use of cell therapy to treat an FSHD animal model. It provides the proof of concept for the potential of PSC-based therapy to improve the dystrophic phenotype induced by DUX4 expression. Pax3-induced mouse ESC-derived myogenic progenitors are able to engraft in the TA of iDUX4pA-HSA mice fed with a dox diet. In the best cases, we obtained more than 600 donor-derived fibers, corresponding to 25% of the muscle area. More importantly, these donor-derived myofibers induce two major functional improvements: the reduction of the extent of fibrosis, and an improvement in muscle force.

As mentioned earlier, the use of PSC-based therapy has shown success in improving the phenotype of mouse models for DMD, LGMD2A, LGMD2D, and LGMD2I^41,44,46,48^. However, the engraftment of WT cells into a dominant MD model has an important distinction to engraftment in recessive models. In a dominant context, it is not a priori clear whether the detrimental factor could be transferred to the newly engrafted donor-derived myofibers. Two studies have previously investigated this issue with contrasting results in different disease models. The first employed FGR1 overexpressing mice, which present a dominant muscle phenotype^58^. The transplantation of mouse ESC-derived myogenic progenitors showed improvement in TA muscle force in males, which are more affected than females^59^. The second study utilized HSA^LR^ mice, a model for the dominant DM^60^. This mouse model carries 250 CUG repeats in the context of the human skeletal actin gene, which leads to the formation of toxic RNA foci in the myonuclei. Mondragon-Gonzalez and colleagues showed that these RNA foci could be transferred to the newly formed donor-derived myofibers, derived from both human and mouse PSC-derived myogenic progenitor transplantation^61^. This transfer resulted in disrupted alternative splicing within donor-derived myofibers^61^. The clinical relevance of this study is difficult to extrapolate, as the HSA^LR^ mouse model shows much higher levels of toxic RNA compared to human DM1, but it is interesting that it presents a case in which a dominant genetic factor impairs the donor-derived WT tissue.

In this manuscript, we have shown that transplantation of PSC-derived myogenic progenitors into TA muscles of iDUX4pA-HSA mice resulted in engraftment in all tested cohorts, with and without CTX pre-injury (Fig. 1), and most importantly, in the context of different pulses and bursts of DUX4 expression (Fig. 2). In the 7 + 14 group, which experienced the most severe DUX4-induced disease phenotype, our results show that donor-derived myofibers ameliorate fibrosis (Fig. 4) and enhance muscle force (Fig. 5). Because transplanted cells also gave rise to donor-derived satellite cells, it is plausible to hypothesize that engraftment will be persistent, as observed in mouse models of DMD and LGMD2I^41,48^. The experiments in which DUX4 expression post-transplantation leads to greater engraftment have obvious relevance to FSHD, in which deterioration is sporadic and continual. One would expect that if an FSHD muscle were transplanted with WT cells, then with each cycle of degeneration, a greater and greater extent of the muscle would be regenerated with WT cells. This is in agreement with our data as we observed the greatest extent of engraftment in the group with the longest period of DUX4-mediated degeneration. It is also possible that transplanted Pax3-induced myogenic progenitors become re-induced by the dox provided in the chow at 3 weeks post-injection (Fig. 2a), resulting in their re-activation/expansion, and ultimately superior engraftment. Our data from the experimental cohort in which mice were treated uninterruptedly with dox show that constant dox treatment in the chow maintains Pax3 expression in transplanted cells, preventing their in vivo differentiation into myofibers. It is plausible that dox re-induction may trigger both DUX4-induced degeneration and Pax3-induced reactivation, but it seems that engraftment size differences observed among different experimental groups are due to DUX4 only. Indeed, the engraftment expansion did not occur in the context of transplantation of Pax3-induced myogenic progenitors into non-DUX4 muscle (Fig. 3a, b and Supplementary Fig. 6a, b). To confirm that enhanced muscle engraftment after the second round of dox treatment was due to effects on the host rather than re-expression of Pax3 in the donor cells, we transplanted WT freshly isolated satellite cells into a cohort of iDUX4pA-HSA mice. This also resulted in donor-derived muscle regeneration that trended toward increased donor contribution with more severe DUX4-mediated degeneration (Fig. 3c, d).

In summary, these findings represent an example of cell therapy in an FSHD model, and demonstrate the benefits of PSC-based myogenic regenerative therapy for FSHD, providing proof of concept for the potential therapeutic application of cell transplantation for autonomous dominant MD.

## Methods

### Cell culture and differentiation

Inducible Pax3-GFP mouse ESCs were cultured in 1:1 ES medium and 2 inhibitors (2i) medium. ES medium is composed of KnockOut^TM^ DMEM (Invitrogen) with 15% FBS (Sigma), 1% penicillin-streptomycin (Invitrogen), 2 mM Glutamax (Gibco), 0.1 mM non-essential amino acids (Gibco), and 0.1 mM β-mercaptoethanol (Gibco). 2i medium consists of neurobasal medium (Invitrogen) and DMEM F12 medium (Invitrogen) supplemented with 0.5% N2 (Life Technologies), 0.5% B27 (Life Technologies), 0.05% BSA (Sigma), 1% penicillin–streptomycin, 150 µM monothioglycerol (MP Biomedicals), 3 µM GSK3β inhibitor (CHIR 990217; Tocris), 1 µM PD 0325901 (Cayman) and 1000 U/ml LIF (Millipore). To facilitate tracking of donor engraftment, ESCs were labeled with a lentiviral vector encoding the fusion protein histone 2B-red fluorescent protein (H2B-RFP; LV-RFP plasmid, Addgene #26001). H2B-RFP plasmid was co-transfected with packaging plasmids Δ8.91 and pVSVG into 293T cells using the LTX transfection reagent (Thermo Fisher Scientific). Lentiviral-containing supernatant was collected 48 h later, filtered, and used to transduce ESCs using the spin infection method (90 min at 2500 rpm at 30 °C). RFP + ESCs were FACS sorted and maintained in ES + 2i medium. ESCs were then plated at a density of 40,000 cells/ml in embryoid bodies (EB) differentiation medium, which consists of IMDM (Invitrogen) supplemented with 15% FBS (Sigma), 1% penicillin/streptomycin (Invitrogen), 2 mM GlutaMAX (Invitrogen), 50 μg/ml ascorbic acid (Sigma-Aldrich), and 4.5 mM monothioglycerol (MP biomedicals). These dishes were cultured on a slowly swirling table rotator (80 rpm) in a cell culture incubator at 37 °C, 5% CO_2_. Pax3-GFP was induced by adding dox (Sigma-Aldrich) on day 3 of EB differentiation (final concentration 1 μg/ml). On day 5, EBs were disaggregated, and stained for 20 minutes with PDGFRα-PE, FLK1-APC, (e-Bioscience). PDGFRα + FLK1 − RFP + myogenic progenitors were sorted using FACSAria II (BD biosciences), plated on gelatin-coated dishes with EB differentiation media supplemented with 1 μg/ml dox and 10 ng/ml mouse basic FGF (bFGF; Peprotech) and maintained 3 passages in culture before transplantation^41,48,62^.

### Satellite cell isolation

Skeletal muscles from mTmG mice^63^ were dissected, chopped with a blade, and digested for 60 min in a 37 °C shaker with 2 mg/ml Collagenase II (Gibco) in DMEM high glucose (Gibco) and 1% penicillin/streptomycin (Invitrogen). Then one volume of PBS was added to the suspension and centrifuged at 500 g for 10 min. The pellet was resuspended twice in a rinsing solution containing 10% horse serum (Hyclone), 1% penicillin/streptomycin (Invitrogen) and 0.1 μM HEPES (Gibco) in Ham’s/F-10 media (Hyclone) and spun down twice at 500 × *g* for 7 min. Then digested tissue was shred with the help of a Pasteur pipette and digested a second time with a solution containing 0.1 mg/ml collagenase II and 0.5 mg/ml Dispase (Gibco) in a rinsing solution incubated for 30 min at 37 °C. The digested solution was homogenized with 16 and 18 G needles, filtered with a 40 µm cell strainer, and centrifuge at 700 g for 7 min. Cells were resuspended in FACS buffer containing 10% FBS (Sigma) and 1% penicillin/streptomycin (Invitrogen) in PBS (Gibco) containing the primary antibodies CD31 and CD45 (both PeCy7-conjugated, eBiosciences), APC-conjugated integrin alpha 7 (itga7) and biotin-Vcam1 (eBiosciences) kept for 20 min in ice then centrifuged at 1000 × *g* for 5 min, and stained with the secondary antibody APC-cy7-conjugated streptavidin for 10 min in ice and centrifuged again at 1000 × *g* for 5 min. Stained cells were resuspended in FACS buffer for sorting using a FACSAria II.

### Mice

All animal studies were performed according to protocols approved by the University of Minnesota Institutional Animal Care and Use Committee. Four-week-old female mice carrying both the iDUX4pA and HSA-rtTA transgenes^22^ were fed with dox chow containing 62.5 mg/kg dox (ENVIGO)^50^. Four-week-old female BL6 and NSG mice were purchased from Jackson Laboratories. mTmG mice^63^, kindly provided by Dr. Bryce Binstadt (University of Minnesota), were used for satellite cell isolation.

### Cell transplantation and muscle collection

TA muscles from iDUX4pA-HSA mice, anesthetized with ketamine/xylazine at 80 mg/kg by intraperitoneal (IP) injection, were injected with 10^6^ myogenic progenitors whereas the contralateral leg received PBS. For satellite cell transplantation, 5000 cells were transplanted. One day prior to transplantation, TA muscles were pre-injured or not with 15 µl of CTX 10 µM (Latoxan). For immunosuppression, recipients received daily IP injections of the immunosuppressant agent tacrolimus (MedChemExpress) at a dose of 5 mg/kg. Treatment started two days before transplantation and ended by the day of euthanasia^41^. TA muscles were collected for engraftment assessment 4–5 weeks later.

### Immunofluorescence staining

Dissected TA muscles were embedded in Tissue-Tek O.C.T. compound (Sakura), and snap frozen on isopentane pre-cooled with liquid nitrogen. In all, 14 µm cryosections were collected on glass slides and conserved at −80 °C. Before staining, muscle cryosections were rehydrated with PBS for 5 min at room temperature (RT). Then fixed with 4% PFA for 30 min at RT, washed with PBS, permeabilized 15 min at RT with 0.3% Triton X100 (Sigma) in PBS, washed again with PBS, blocked for 30 min with 3% BSA (Sigma), and incubated overnight at 4 °C with primary antibodies. Primary antibodies included RFP (rabbit 1:500, ab62341 Abcam), dystrophin (Dys, mouse 1:20, DYS1-CE Leica), PCM1 (rabbit 1:500, HPA023370 Millipore Sigma), laminin α-2 (Lam, rat 1:200, Sc-59854 Santa Cruz), Pax7 (mouse 1:10, DSHB), and Col VI (rabbit 1:100, 17023-1-AP Thermo Fisher Scientific). The next day, cryosections were rinsed with PBS and incubated with Alexa Fluor (Thermo Fisher Scientific) secondary antibodies and 4,6-Diamidino-2-phenylindole (DAPI, Santa Cruz) for 1 hr at RT. After three PBS washes, sections were dried and mounted with Prolong Gold with DAPI (Invitrogen). Slides were analyzed by confocal microscopy (NikonNiE C2 upright confocal microscope). Image processing and quantification were performed with Fiji software. Merge images of RFP, Dys, and DAPI were used to quantify donor-derived engraftment. For donor-derived satellite cell engraftment, merge images of RFP, Pax7, laminin α-2, and DAPI were used to quantify the frequency of Pax7 + /RFP + cells. A total of 11-12 cryosections were analyzed. For Pax3 staining, we used an antigen retrieval method, slides were fixed with 4% PFA for 30 min at RT, followed by antigen retrieval in citrate Buffer (1.8 mM Citric Acid and 8.2 mM Sodium Citrate in water). Slides were boiled in a Coplin jar for 30 min, then rinsed with cold tap water for 10 min, washed twice with PBS, 5 min each. After antigen retrieval, sections were incubated with 3% H2O2 in PBS for 5 min, and washed twice with PBS, 5 min each. Sections were blocked with 3% BSA (Sigma), then incubated overnight at 4 °C with primary antibodies: RFP (rabbit 1:500, ab62341 Abcam) and Pax3 (mouse 1:100, MAB2457 R&D systems). The next day, cryosections were rinsed with PBS and incubated with Alexa Fluor (Thermo Fisher Scientific) secondary antibodies and DAPI (Santa Cruz) for 1 h at RT. After three PBS washes, sections were dried and mounted with Prolong Gold with DAPI (Invitrogen)^64^.

### H&E and Masson’s trichrome staining

H&E staining was performed using the SelecTech kit (Leica Biosystems). Masson’s trichrome staining was utilized to detect collagen content (Masson’s Trichrome Stain Kit, 25088 Polysciences, Inc.). Image processing and quantification were performed with Fiji software using the complete muscle section. The area of collagen deposition was reported as the total section area.

### In situ force measurement assay

Measurements of TA muscle force were performed using a 3-in-1 animal system (Aurora Scientific). Mice were maintained anesthetized with isoflurane TA muscle was exposed, the knee stabilized with a knee pin and clamp, and the TA distal tendon was cut and connected to the force transducer with a silk loop. Electrodes were positioned under the TA. The optimal length (*L*_0_) was determined using a series of rectangular unipolar pulses of 0.2 ms at different muscle base tensions to stimulate the TA muscles. Then, the maximal tetanic force (*F*_0_) was recorded at 150 Hz using the same rectangular unipolar pulses of 0.2 ms for 300 ms. The cross-sectional area (CSA) and the specific force (s*F*_0_) were calculated after the measurement of muscle optimal length and weight for each muscle^65^.

### Statistical analysis

Differences between the two groups were assessed by using the Student’s *t* test for independent samples. *p* values < 0.05 were considered significant. Statistical analyses were performed using Prism Software (GraphPad).

### Reporting summary

Further information on research design is available in the Nature Research Reporting Summary linked to this article.

## Supplementary information

Supplementary Figures

REPORTING SUMMARY

## Data Availability

The data supporting the findings in this study are available within the article and its Supplementary Information file. Any raw data generated and analyzed during this study are available from the corresponding author at request. Reasonable requests of unprocessed images and raw data files used for quantifications presented in the article or supplementary information may be submitted via email to perli032@umn.edu.
