# Supplementary material for: Transplantation of PSC-derived myogenic progenitors counteracts disease phenotypes in FSHD mice
Source: NPJ Regen Med. 2022 Sep 2;7:43. doi: 10.1038/s41536-022-00249-0 (PMC9440030; doi:10.1038/s41536-022-00249-0)
Supplement: Supplementary file 1 — Supplementary Figures [file 41536_2022_249_MOESM1_ESM.pdf]

Supplementary Figure 1

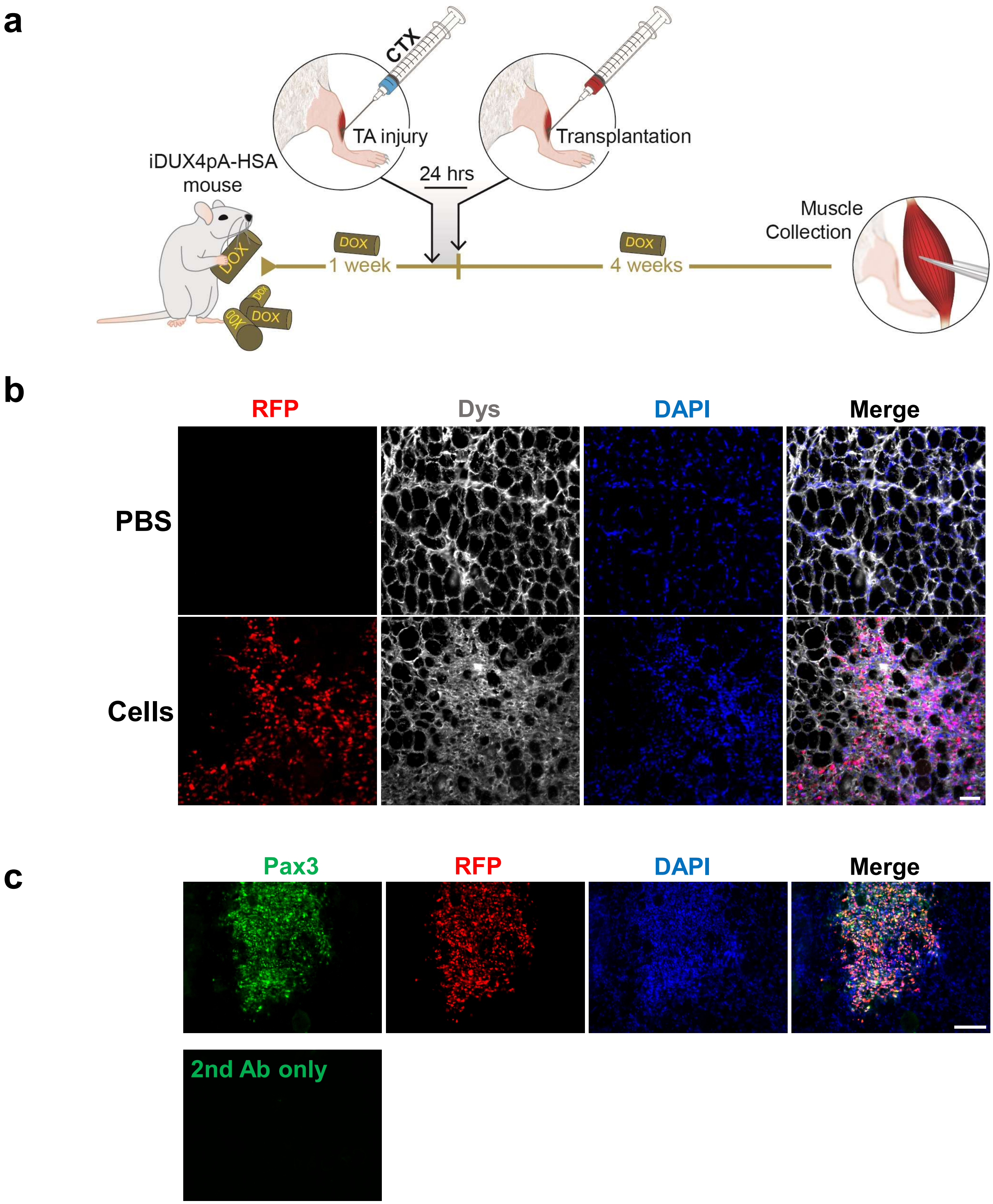

Supplementary Figure 1. Engraftment analysis in transplanted iDUX4pA-HSA mice that had been exposed to constant dox treatment. a) Outline of experimental design. **b)** Representative images show immunostaining for RFP (in red) and Dys (in gray) in TA muscles of iDUX4pA-HSA mice that had been injected with PBS (upper panel) or cells (lower panel) and maintained on dox treatment throughout the study. DAPI stained nuclei (in blue). Scale bar is 50 µm. **c)** Representative images show immunostaining for Pax3 (in green), RFP (in red), DAPI (in blue), and merge. Lower left panel shows control staining using mouse secondary antibody alone (2nd Ab only, in green), Scale bar is 100 µm.

Supplementary Figure 2

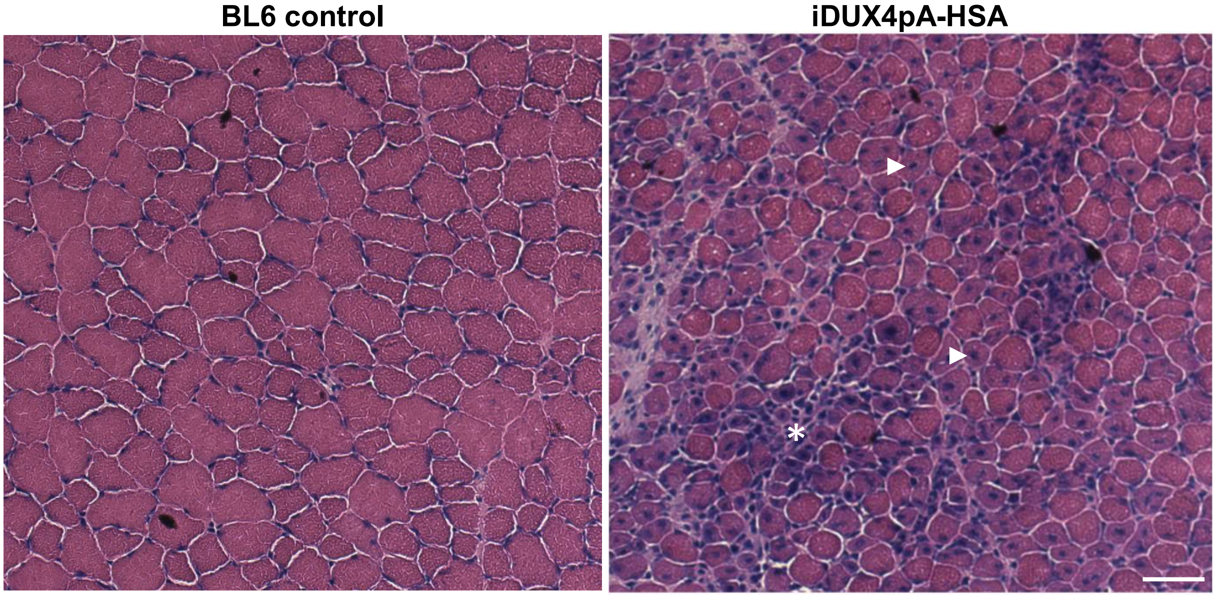

Supplementary Figure 2. Histological characterization of iDUX4pA-HSA mice after 7 days of dox treatment. Representative images show H&E staining of TA muscle cryosections obtained from 5-week-old BL6 sibling control and iDUX4pA-HSA mice. Arrows indicate centrally located nuclei and asterisks denote the presence of infiltrating mononuclear cells. Scale bar is 50 μm.

Supplementary Figure 3

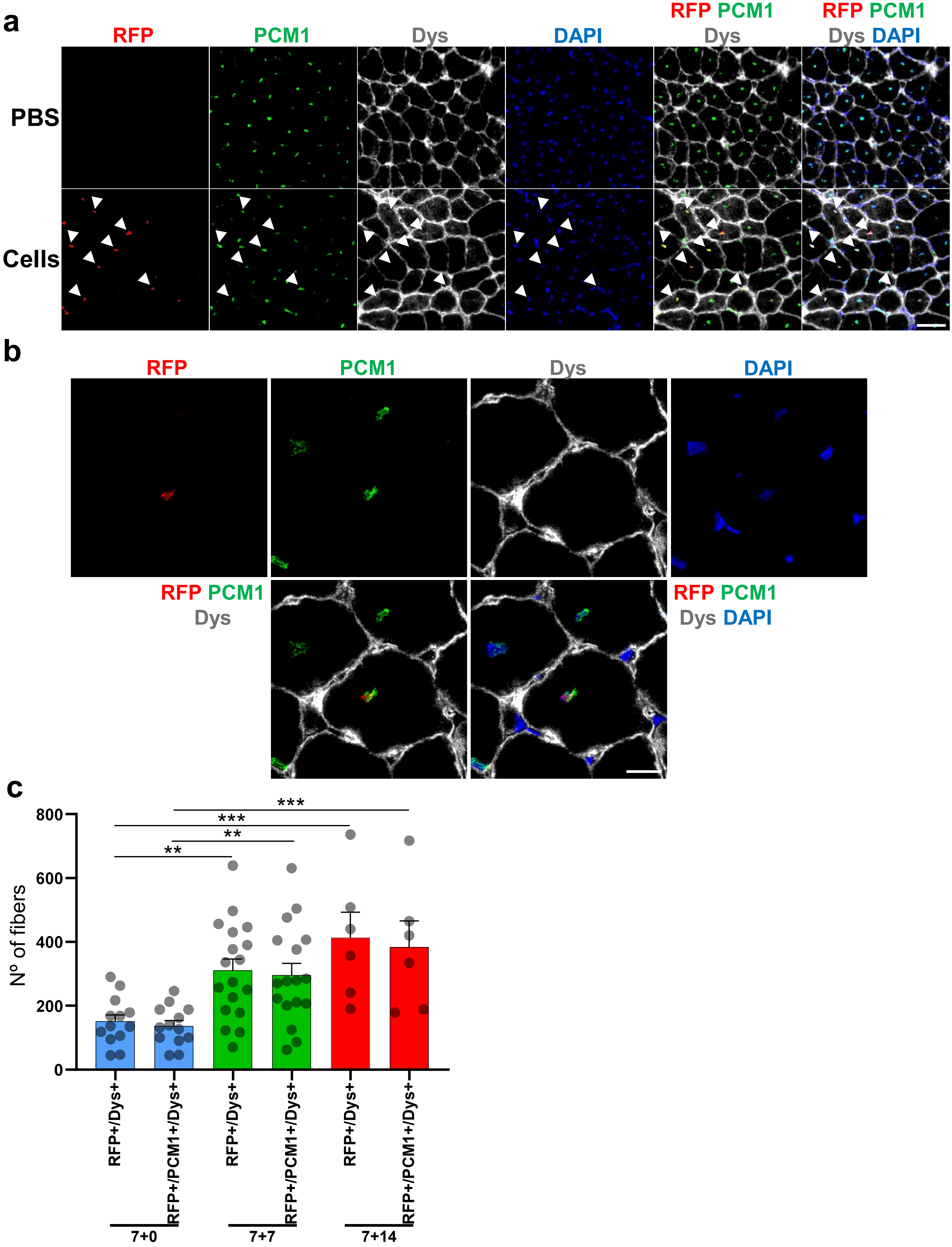

Supplementary Figure 3

Supplementary Figure 3. PCM1 staining and engraftment validation for the groups 7+0, 7+7 and 7+14. a) Representative images show immunostaining for RFP (in red), PCM1 (in green) and Dys (in gray) in TA muscles of iDUX4pA-HSA mice that had been injected with PBS (upper panel) or cells (lower panel) from the group 7+0. DAPI stained nuclei (in blue). Arrowheads show RFP+/PCM1+ nuclei. Scale bar is 50 µm. **b)** Representative confocal images show an RFP and PCM1 positive nuclei in TA muscles of iDUX4pA-HSA transplanted with cells from the group 7+0. DAPI stained nuclei (in blue). Scale bar is 10 µm. **c)** Graph shows quantification of engraftment (from Fig. 2) obtained with our 3 different dox regimen. Quantification has been performed by 2 different ways: RFP+/Dys+ donor-derived myofibers (from Fig. 2), and RFP+/PCM1+/Dys+ donor-derived myofibers. Data are shown as mean ± SEM (n=6 for -CTX, n=4 for +CTX). Data are shown as mean ± SEM (for RFP+/Dys+ n=13 for 7+0, n=18 for 7+7, and n=6 for 7+14; for RFP+/PCM1+/Dys+ n=13 for 7+0, n=17 for 7+7, and n=6 for 7+14). **$p < 0.01$, ***$p < 0.001$ by the Student's t-test.

Supplementary Figure 4

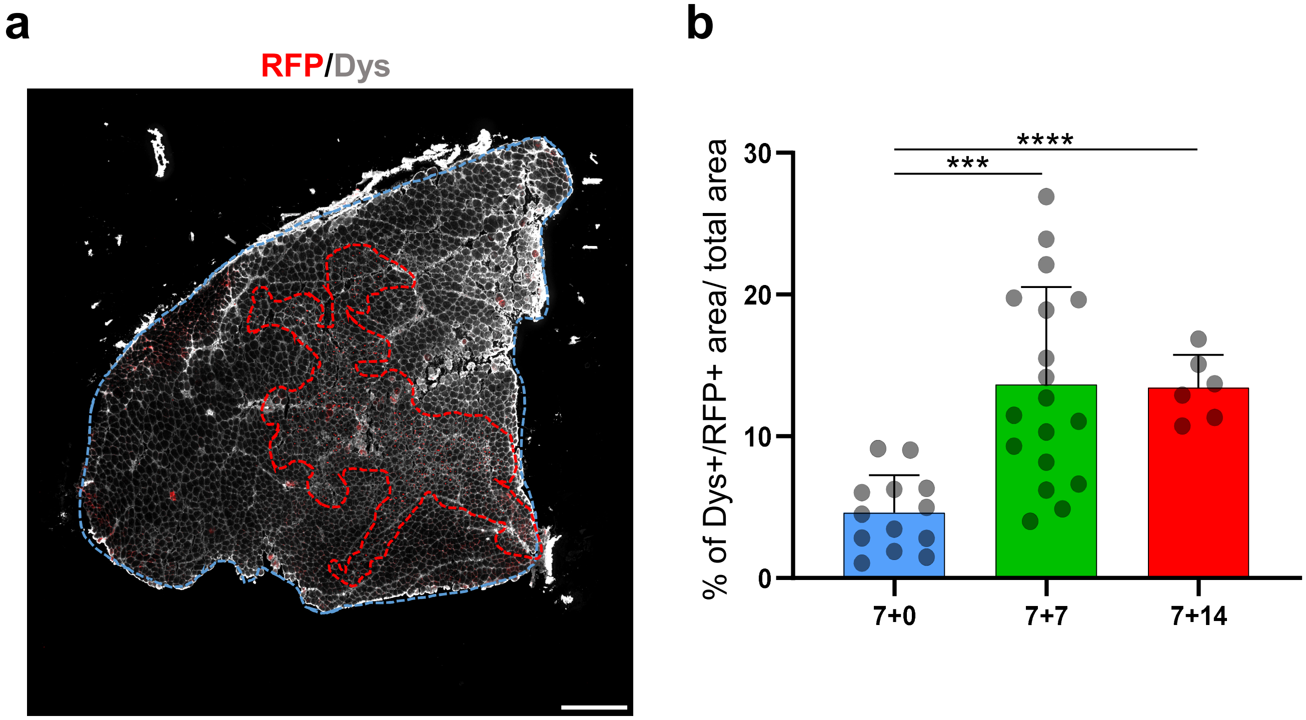

Supplementary Figure 4. Percentage of engrafted area in transplanted TA muscles of iDUX4pA-HSA mice. a) Representative image displays the size of engrafted area (marked in red) compared to the total cryosection area (marked in blue), as evidenced by the immunofluorescence staining for RFP (in red) and Dys (in gray). Scale bar is 500 µm. **b**) Quantification of the percentage of engrafted area compared to the total cryosection area in the 3 experimental cohorts of iDUX4pA-HSA mice outlined in Fig. 2a: 7+0, 7+7, and 7+14. Data are shown as mean ± SEM (n=13 for 7+0, n=18 for 7+7 and n=6 for 7+14). ***$p < 0.001$, ****$p < 0.0001$ by the Student's t-test.

Supplementary Figure 5

a

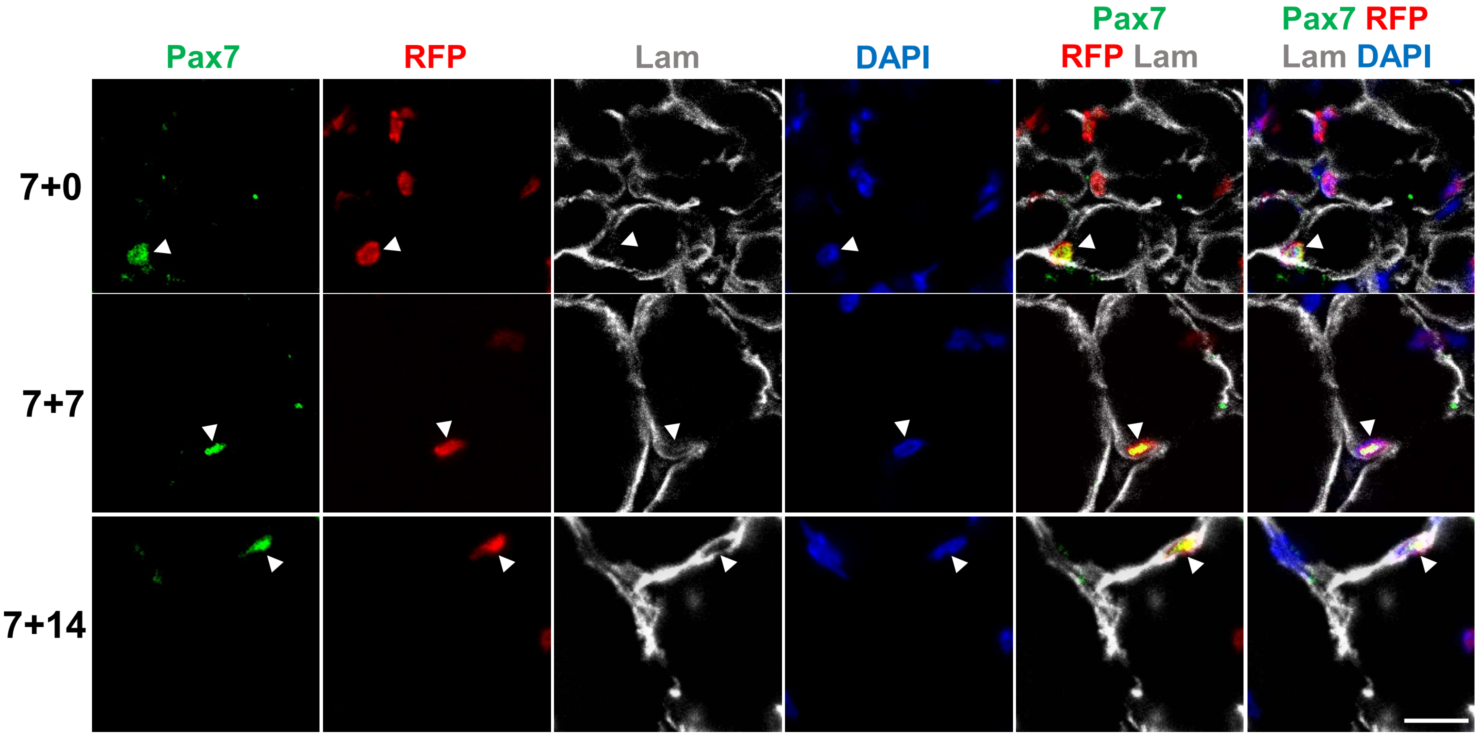

b

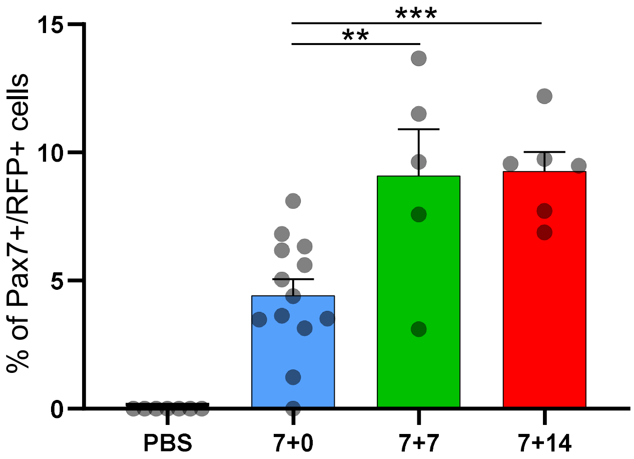

Supplementary Figure 5. Quantification of satellite cell engraftment in transplanted iDUX4pA-HSA mice. a) Representative images show donor-derived satellite cell engraftment for the 3 experimental groups, as indicated by the presence (arrowhead) of a cell under the basal lamina (Lam in gray), co-expressing RFP (in red) and Pax7 (in green). DAPI in blue stains nuclei. Scale bar is 10 µm. **b)** Quantification of donor-derived satellite cell engraftment, as shown by the percentage of Pax7+/RFP+ cells in the 3 experimental cohorts of iDUX4pA-HSA mice: 7+0, 7+7, and 7+14. Data are shown as mean ± SEM (n=13 for 7+0, n=5 for 7+7 and n=6 for 7+14). **$p < 0.01$, ***$p < 0.001$ by the Student's t-test.

Supplementary Figure 6

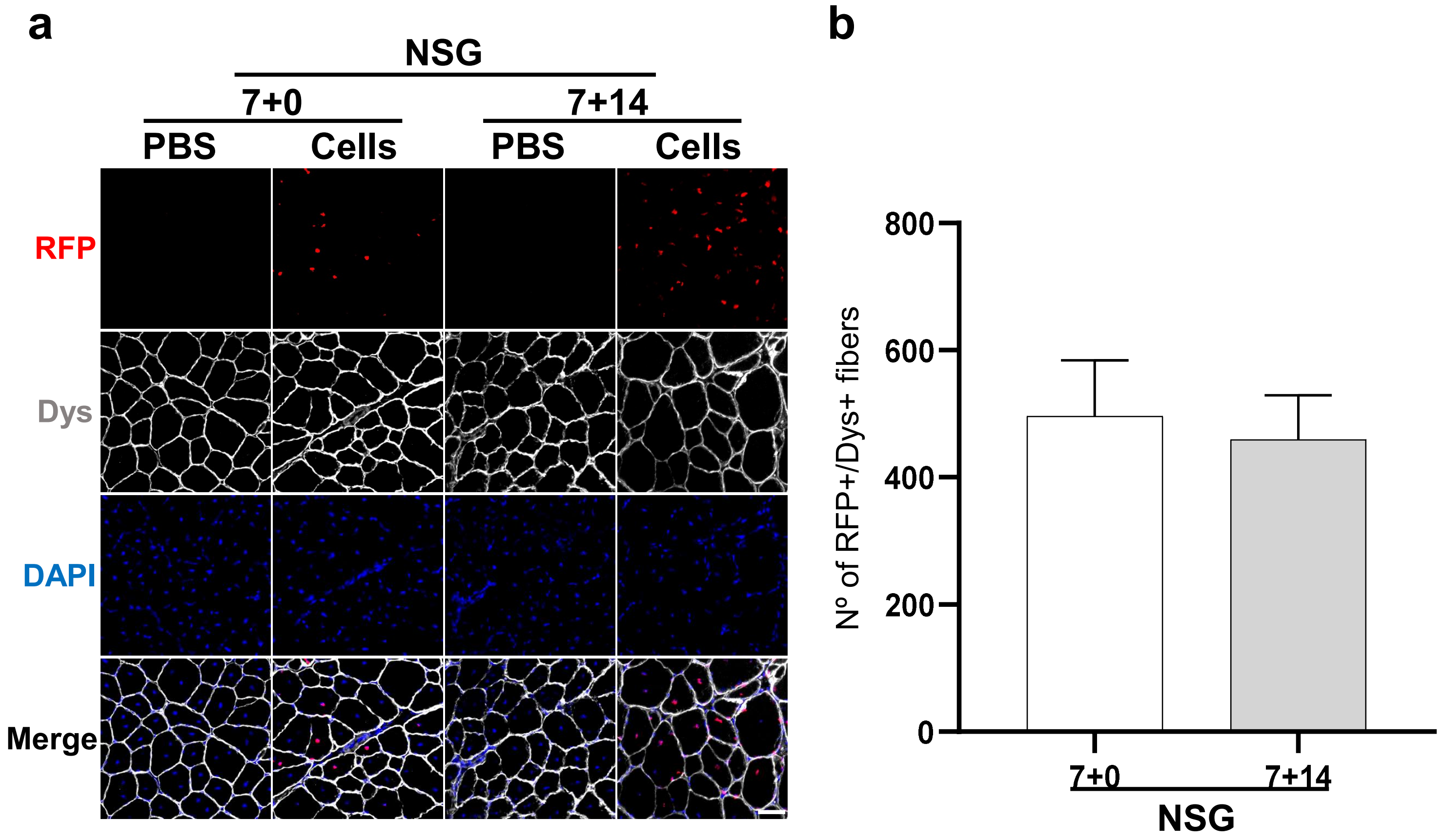

Supplementary Figure 6. Engraftment assessment in NSG mice upon dox diet. a) Representative images show immunostaining for RFP (in red), and Dys (in grey) NSG TA muscles injected with PBS or cells and fed with dox like the iDUX4pA-HSA mouse groups 7+0 and 7+14. DAPI stained nuclei (in blue). Scale bar is 50 µm. **b)** Graph shows quantification of engraftment based on the number of RFP+/Dys+ myofibers in NSG cell injected TAs from the group 7+0 and 7+14. Data are shown as mean ± SEM (n=7 for 7+0 and n=7 for 7+14 for NSG mice).

Supplementary Figure 7

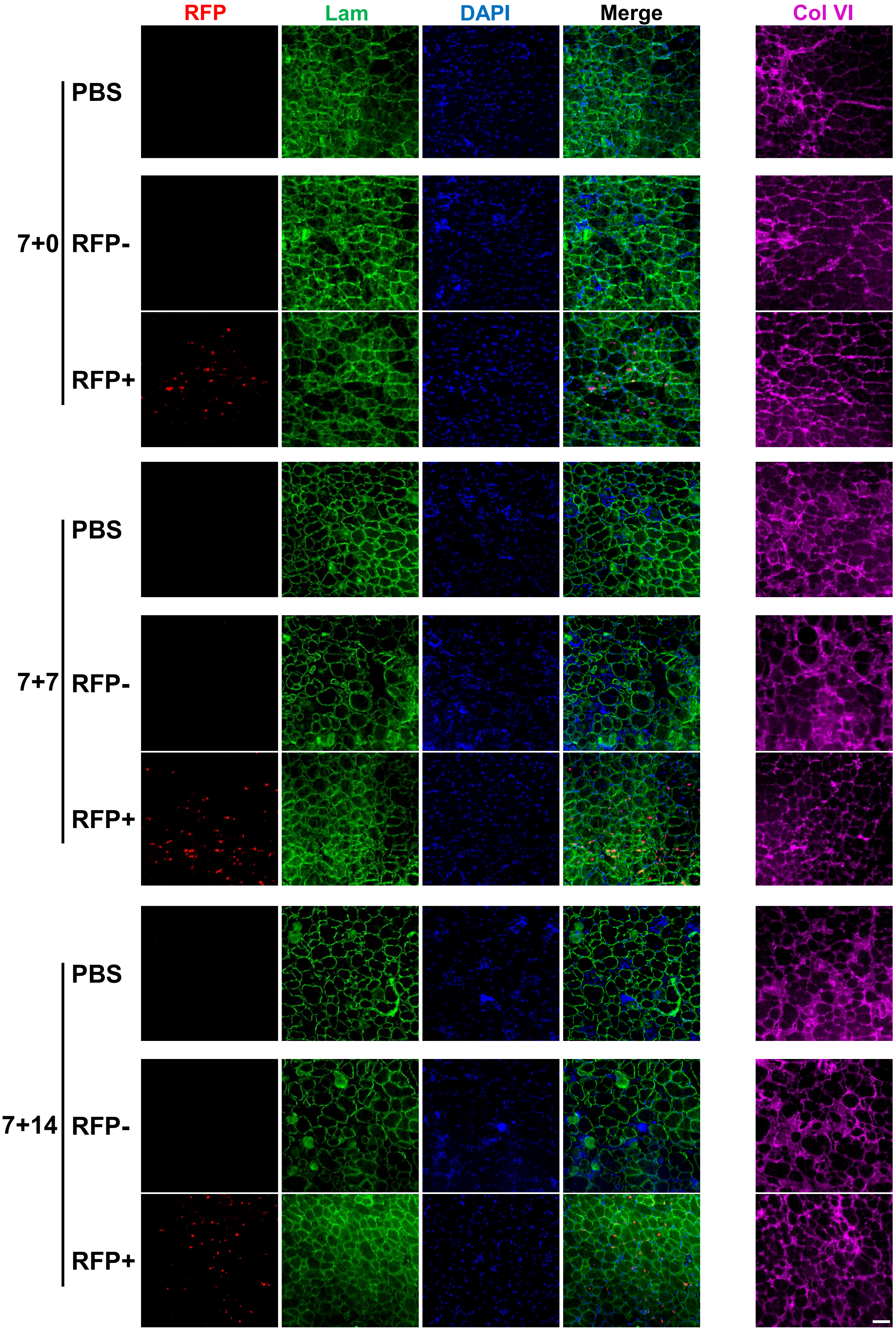

Supplementary Figure 7. High magnification of collagen deposition in engrafted compared to non-engrafted area. Representative images show immunostaining for RFP (in red),and Lam (in green) of iDUX4pA-HSA mouse TA muscles injected with PBS or cells in area showing donor-derived myofiber (RFP+) or not (RFP-) from the 3 different dox regimen (7+0, 7+7, 7+14). Col VI (in purple) immunostaining has been performed on the consecutive slide and show the same representative area. DAPI stained nuclei (in blue). Scale bar is 50 µm.
