# Supplementary material for: Transplantation of PSC-derived myogenic progenitors counteracts disease phenotypes in FSHD mice
Source: NPJ Regen Med. 2022 Sep 2;7:43. doi: 10.1038/s41536-022-00249-0 (PMC9440030; doi:10.1038/s41536-022-00249-0)
Supplement: Supplementary file 2 — REPORTING SUMMARY [file 41536_2022_249_MOESM2_ESM.pdf]

nature portfolio

Corresponding author(s): Rita C.R. Perlingeiro

Last updated by author(s): Jul 22, 2022

Reporting Summary

Nature Portfolio wishes to improve the reproducibility of the work that we publish. This form provides structure for consistency and transparency in reporting. For further information on Nature Portfolio policies, see our Editorial Policies and the Editorial Policy Checklist.

Statistics

For all statistical analyses, confirm that the following items are present in the figure legend, table legend, main text, or Methods section.

n/a	Confirmed	
☐	☒	The exact sample size (*n*) for each experimental group/condition, given as a discrete number and unit of measurement
☐	☒	A statement on whether measurements were taken from distinct samples or whether the same sample was measured repeatedly
☐	☒	The statistical test(s) used AND whether they are one- or two-sided<br>*Only common tests should be described solely by name; describe more complex techniques in the Methods section.*
☒	☐	A description of all covariates tested
☒	☐	A description of any assumptions or corrections, such as tests of normality and adjustment for multiple comparisons
☐	☒	A full description of the statistical parameters including central tendency (e.g. means) or other basic estimates (e.g. regression coefficient) AND variation (e.g. standard deviation) or associated estimates of uncertainty (e.g. confidence intervals)
☒	☐	For null hypothesis testing, the test statistic (e.g. *F*, *t*, *r*) with confidence intervals, effect sizes, degrees of freedom and *P* value noted<br>*Give P values as exact values whenever suitable.*
☒	☐	For Bayesian analysis, information on the choice of priors and Markov chain Monte Carlo settings
☒	☐	For hierarchical and complex designs, identification of the appropriate level for tests and full reporting of outcomes
☒	☐	Estimates of effect sizes (e.g. Cohen's *d*, Pearson's *r*), indicating how they were calculated

Our web collection on statistics for biologists contains articles on many of the points above.

Software and code

Policy information about availability of computer code

Data collection	Nikon confocal microscope: NIS element software, Zen upright microscope: Zen 2.3 pro software, Aurora 3 in 1: 610A DMC LabBook software.
Data analysis	Fiji (Image J 2.1.0/1.53c Java 1.8.0_172), GraphPad Prism 9.1.2, Zen 3.4 blue edition software, Aurora: ASI 611A Dynamic muscle Analysis v5.501 software.

For manuscripts utilizing custom algorithms or software that are central to the research but not yet described in published literature, software must be made available to editors and reviewers. We strongly encourage code deposition in a community repository (e.g. GitHub). See the Nature Portfolio guidelines for submitting code & software for further information.

Data

Policy information about availability of data

All manuscripts must include a data availability statement. This statement should provide the following information, where applicable:

- Accession codes, unique identifiers, or web links for publicly available datasets
- A description of any restrictions on data availability
- For clinical datasets or third party data, please ensure that the statement adheres to our policy

The datasets used and/or analyzed during the current study are available from the corresponding author on reasonable request. Materials used in this study are commercially available.

Field-specific reporting

Please select the one below that is the best fit for your research. If you are not sure, read the appropriate sections before making your selection.

☒ Life sciences ☐ Behavioural & social sciences ☐ Ecological, evolutionary & environmental sciences

For a reference copy of the document with all sections, see nature.com/documents/nr-reporting-summary-flat.pdf

Life sciences study design

All studies must disclose on these points even when the disclosure is negative.

Sample size	Sample size was determined to insure the robustness of the results observed with 4 to 18 mice per experimental groups and correlate with the breeding of the iDUX4pA-HSA transgenic mice.
Data exclusions	no data where excluded from the analysis.
Replication	To verify the reproducibility of experimental finding we used a large n of mice and reproduce the experiment with different animal cohorts when possible.
Randomization	The iDUX4pA-HSA mice were allocated randomly to the different groups present in this study.
Blinding	The different quantification and analysis were performed blindly between the different experimental groups also between PBS vs Cells.

Reporting for specific materials, systems and methods

We require information from authors about some types of materials, experimental systems and methods used in many studies. Here, indicate whether each material, system or method listed is relevant to your study. If you are not sure if a list item applies to your research, read the appropriate section before selecting a response.

Materials & experimental systems

n/a	Involved in the study	
☐	☒	Antibodies
☐	☒	Eukaryotic cell lines
☒	☐	Palaeontology and archaeology
☐	☒	Animals and other organisms
☒	☐	Human research participants
☒	☐	Clinical data
☒	☐	Dual use research of concern

Methods

n/a	Involved in the study	
☒	☐	ChIP-seq
☒	☐	Flow cytometry
☒	☐	MRI-based neuroimaging

Antibodies

Antibodies used	anti-RFP: ab62341 Abcam, anti-dystrophin: DYS1-CE Leica, anti-laminin α2: Sc-59854 Santa Cruz, anti-Pax7: DSHB, anti-col VI: 17023-1-AP Thermo Fisher Scientific and anti-Pax3: MAB2457 R&D systems, anti-PCM1 :HPA023370 Millipore Sigma.
Validation	All these antibodies are commercially available and have been widely used and characterized by the scientific community.

Eukaryotic cell lines

Policy information about cell lines

Cell line source(s)	The cells used in this study are doxycycline inducible PAX3-GFP mouse embryonic stem cells
Authentication	Cells where identified using PAX3 expression upon doxycycline
Mycoplasma contamination	This cell line is regularly tested for mycoplasma contamination and appears negative.
Commonly misidentified lines (See ICLAC register)	not applicable

Animals and other organisms

Policy information about studies involving animals; ARRIVE guidelines recommended for reporting animal research

Laboratory animals	8-week-old female iDUX4pA-HSA mice and 6-week-old male and female C57BL/6 mice and 6-week-old female NSG mice.
Wild animals	the study did not involved wild animal
Field-collected samples	the study did not involved samples collected from the field
Ethics oversight	All animal studies were performed according to protocols approved by the University of Minnesota Institutional Animal Care and Use Committee.

Note that full information on the approval of the study protocol must also be provided in the manuscript.
